# Editorial: The role of epithelial-derived cytokines in airway disease

**DOI:** 10.3389/falgy.2026.1797785

**Published:** 2026-02-24

**Authors:** Hector Ortega, Kian Fan Chung

**Affiliations:** 1 Prana Therapies, San Diego, CA, United States; 2 Imperial College London, National Heart & Lung Institute, London, United Kingdom

**Keywords:** alarmin cytokines, asthma, biologics, bispecfic antibody, COPD, respiratory diseases

In 2005, Dr. Joost Oppenheim introduced the term *alarmin* to describe endogenous molecules that function as early warning signals for the immune system by indicating tissue injury or cellular stress (1). These molecules rapidly mobilize innate and adaptive immune responses following diverse triggers such as infection or trauma. Acting as early responders, alarmins activate immune cells and promote the production of proinflammatory cytokines that shape downstream inflammatory cascades.

The articles included in the Research Topic *Role of Epithelial-Derived Cytokines in Airway Diseases* collectively examine the multifaceted biology of alarmins and their impact on airway health and disease. Bondi et al. describe the emerging role of microplastics (MPs) as environmental contaminants capable of inducing epithelial inflammation and damage. Their review critically evaluates current evidence on MP–epithelium interactions, highlighting both mechanistic insights and methodological limitations. Inhaled MPs can deposit throughout the respiratory tract, interact with the epithelial surface, and trigger inflammatory, oxidative, and structural alterations relevant to disease onset and progression. MPs induce epithelial stress responses—including oxidative stress, activation of inflammatory signaling, and disruption of junction-related proteins—which may compromise mucociliary function. Persistent epithelial injury may activate the NLRP3 inflammasome and drive TGF-β1–mediated fibroblast activation and extracellular matrix deposition, contributing to sustained inflammation and airway remodeling (2). MPs therefore represent an underrecognized but potentially important environmental factor influencing chronic airway inflammation.

Another relevant trigger of alarmins is explored by Sang et al. who focus on the immune consequences of rhinovirus infection. Rhinovirus, a common respiratory pathogen and frequent exacerbating factor in asthma (3), is associated with distinct shifts in cytokine expression during infection. Their review highlights increase in both T2-high and T2-low cytokines, as well as elevated alarmins, during rhinovirus-induced immune responses. Inhibition studies suggest that modulating these cytokine pathways may reduce the intensity of virus-driven inflammation, offering potential avenues for therapeutic intervention in diseases exacerbated by rhinovirus.

Parnham et al. examine the role of epithelial cells in sustaining immune defense in the airways. The airway epithelium functions not only as a physical barrier but also as a dynamic regulator of immune responses. Among its key mediators are collectins, particularly surfactant protein-D (SP-D) and its related molecule SP-A, which bind pathogens, apoptotic cells, and allergens, promoting phagocytosis while preventing excessive Th2-driven inflammation (4). Their review discusses how epithelial dysfunction in COPD contributes to chronic inflammation and impaired host defense, thereby increasing susceptibility to infection. Insights into epithelial-immune interactions and collectin biology may inform therapeutic strategies aimed at restoring epithelial integrity, bolstering immune defense, and advancing precision-medicine approaches.

Pham and Kim provide a detailed overview of epithelial-derived cytokines in severe asthma, organizing them into several functional groups: the alarmins IL-25, IL-33, and TSLP; proinflammatory cytokines including IL-1, IL-6, and TNF-α; chemokines such as CCL2 and CCL5; and antiviral cytokines including IFN-α, IFN-β, and IFN-λ. Alarmins, released rapidly in response to epithelial injury, initiate immune cascades by activating dendritic cells, T2 innate lymphoid cells, and eosinophils. Proinflammatory cytokines amplify inflammatory signals, while chemokines guide immune cells to sites of tissue damage. The airway epithelial barrier, composed of ciliated, basal, and secretory cells, forms the first line of defense against inhaled environmental exposures. Tight junction proteins, including zonula occludens-1, occludin, and claudin, maintain barrier integrity and protect against airborne allergens, pollutants, and pathogens (5). Ciliated cells coordinate mucociliary clearance, basal cells support epithelial regeneration, and secretory cells produce mucins that form the protective mucus layer. These features highlight the critical importance of mucosal integrity in respiratory diseases.

Pelaia et al. review the central role of TSLP in both T2-high and T2-low asthma. Their work emphasizes evidence supporting TSLP as a key therapeutic target for disrupting epithelial-driven inflammatory pathways. Clinical trials and real-world studies show that blocking TSLP with tezepelumab, the only approved anti-TSLP to date, reduces exacerbations across a broad range of asthma phenotypes (6). A systematic review and meta-analysis by Orzołek et al. reported significantly higher TSLP concentrations in blood, bronchial biopsies, and bronchoalveolar lavage fluid in asthma compared with controls. Variability observed in induced sputum, exhaled breath condensate, and nasal samples underscores ongoing challenges in the standardization of biomarker collection protocols. Nonetheless, the available evidence supports continued efforts to characterize TSLP-related biomarkers and their utility in personalized therapy.

Finally, Komori and Ortega emphasize the importance of understanding compensatory interactions among the alarmins TSLP, IL-33, and IL-25 in a complex immune environment. Given their overlapping and sometimes redundant functions, elucidating the regulatory mechanisms and tissue-specific expression patterns of these cytokines is essential for optimizing therapeutic strategies. Since alarmins function upstream of canonical inflammatory mediators such as IL-4, IL-5, and IL-13, targeting alarmin pathways may provide broader and more comprehensive immunomodulation than therapies directed at downstream cytokines alone. Multispecific antibodies capable of inhibiting multiple alarmins simultaneously may offer enhanced clinical benefit, particularly for patients with heterogeneous disease drivers or incomplete responses to monotherapy. As clinical development in this area rapidly expands, emerging data will help determine further the full therapeutic potential of targeting alarmins across airway diseases. The field is moving toward more refined biologics—including advanced monospecific and multispecific agents—with the goal of improving clinical outcomes through deeper modulation of epithelial-derived inflammatory pathways.
